# Tourette syndrome research highlights from 2017

**DOI:** 10.12688/f1000research.15558.1

**Published:** 2018-07-23

**Authors:** Andreas Hartmann, Yulia Worbe, Kevin J. Black

**Affiliations:** 1 Sorbonne University, National Reference Centre for Tourette Disorder, Pitié-Salpêtrière Hospital, Paris, France; 2 Department of Physiology, Saint-Antoine Hospital, Paris, France; 3 Psychiatry, Neurology, Radiology, and Neuroscience, Washington University School of Medicine, St. Louis, MO, 63110-1093, USA

**Keywords:** Tourette syndrome, tic disorders, review, natural history, etiology, pathophysiology

## Abstract

This is the fourth yearly article in the Tourette Syndrome Research Highlights series, summarizing research from 2017 relevant to Tourette syndrome and other tic disorders. The authors briefly summarize reports they consider most important or interesting. The highlights from 2018 article is being drafted on the Authorea online authoring platform, and readers are encouraged to add references or give feedback on our selections using the comments feature on that page. After the calendar year ends, the article is submitted as the annual update for the Tics collection on F1000Research.

## Introduction

This article is meant to disseminate recent scientific progress on Gilles de la Tourette Syndrome (TS).

## Methods

We searched PubMed from time to time using the search strategy “("Tic Disorders"[MeSH] OR Tourette NOT Tourette[AU]) AND 2017[PDAT] NOT 1950:2016[PDAT]”. On 06 July 2018 this search returned 212 citations. Colleagues also recommended articles, and we attended medical conferences. We selected material to be discussed in this review subjectively, guided by our judgment of possible future impact on the field.

## Results

A number of tic experts contributed to a review article on TS ( Robertson *et al.*, 2017).

### Phenomenology and natural history

Schaefer *et al.* (2017) describe 16 people with TS who had experienced a clinical remission or marked improvement of more than 1 year’s duration, followed by symptomatic worsening as adults, leading them again to seek treatment. On average the “latent period” (the absence or substantial reduction in tics) had lasted 16 years. Seven of them had worse tics when returning for care than they recalled as children. New substance use was reported as a trigger for exacerbation in 5 patients. This report strengthens evidence that even long-lasting symptomatic improvement in tic disorders (TDs) may not always be permanent, and that in fact the typical course of TS “is one of occasional recurrences of mild tics throughout adult life” ( Bruun & Budman, 1997); see also ( Black *et al.*, 2016; Shapiro *et al.*, 1988, p. 188; Stárková, 1990; Singer, 2006).

Regarding natural course and history, the fate of non-tic symptoms in TS has remained less well explored. A large Danish study reported follow-up data 6 years after enrolling 314 children and teenagers with TS, assessing tics and comorbidities (mainly obsessive–compulsive disorder (OCD) and Attention-Deficit/Hyperactivity Disorder (ADHD); N=226 at follow-up) ( Groth *et al.*, 2017). Most patients’ tics improved over time, but almost a quarter of those over age 16 still had severe tics, and only a sixth had no tics. The severity of OCD and ADHD declined significantly during adolescence, suggesting a shift towards so-called “pure” TS with age. Furthermore, the authors expected tic-related impairment to improve with an age-related decline in tic frequency and severity, but surprisingly the impairment score did not reflect the improvement in tics.

A report on 606 patients with a movement disorder starting in childhood produced an estimate for tic onset of 7.4 ± 3.8 years with a mean delay to diagnosis of 9.9 ± 11 years ( Bäumer *et al.*, 2016).

***Epidemiology.*** New and important findings this year involve the previously unappreciated risk of death in TDs. Meier *et al.* (2017) demonstrated that mortality rates are elevated in TS and other TDs, with or without comorbidities. In a very large epidemiological study, TDs in adults were associated with a four-fold higher risk of suicide, with the risk not explained by other psychiatric illness such as major depression ( Fernández de la Cruz *et al.*, 2017b). These researchers analyzed 7736 TS/chronic tic disorder (CTD) cases in the Swedish National Patient Register over a 44-year period (1969–2013) and compared them with control subjects from the general population. An increased risk of both suicide and attempted suicide was observed in TS/CTD patients, which was not solely dependent on psychiatric comorbidities. Tics that persisted beyond young adulthood significantly predicted completed suicide. Thus, TS/CTD is a serious medical condition that requires careful monitoring of suicidality. The same group also found a 7- to 10-fold higher risk of completed suicide in people with OCD, even after controlling for comorbid diagnoses ( Fernández de la Cruz *et al.*, 2017a).

Martino *et al.* (2017c) review screening instruments and rating scales for TDs; see also ( Augustine *et al.*, 2017; Martino & Pringsheim, 2017).

***Transient environmental effects on tic severity.*** A study of 45 children with TS supported the typical antecedent–behavior–consequence behavioral psychology model ( Eaton *et al.*, 2017). Specifically, consequences of tics, “such as receiving accommodations or attention from others,” explained significantly more variance in tic severity than did the child’s level of separation anxiety, though the latter was also a significant factor. This study provides supportive evidence for the approach taken by “CBIT-Jr,” a behavior therapy designed for younger children with TS ( Piacentini *et al.*, 2015).

***Other.*** Non-tic symptoms in TS are reviewed by Martino *et al.* (2017b). Lee *et al.* (2017) used Taiwan’s National Health Insurance Research Database to compare 1124 newly diagnosed TS patients to controls in a 1:3 match. Sleep disorders were twice as common in TS, and remained significantly higher in TS after accounting for anxiety disorders, which were the comorbid conditions associated with the highest risk. In a sample of 811 TS subjects recruited for a genetics study, hair pulling (3.8%) and skin picking (13.0%) disorders by DSM-5 were surprisingly common ( Greenberg *et al.*, 2018).

Autism spectrum disorders (ASD) comprise an underexplored comorbidity of TD. There are few epidemiological studies on the subject but the prevalence of ASD in children with TDs is estimated at 20% ( Khalifa & von Knorring, 2006). In a large study including patients with TD (n=535) and their family members (n=234), Darrow *et al.* (2017a) used the Social Responsiveness Scale Second Edition (SRS) to characterize ASD symptoms, and compared them to historical ASD samples. SRS scores in participants with TD were similar to those observed in other clinical samples but lower than in ASD samples. This is mostly but not entirely explained by elevations in the RRB (restricted interests and repetitive behaviors) subscale, which may be indicating tics rather than other stereotypic movements. The presence of OCD was associated with higher scores on the social cognition and RRB subscales. Complex tics and OCD symptoms (repetitive behaviors) can also be hard to discriminate from core ASD symptoms, especially those related to social communication.

### Etiology

A national database study found that parents of children with chronic tics had significantly higher rates of psychiatric illness: more than twice as high in mothers and 39% higher in fathers ( Leivonen *et al.*, 2017). The maternal risk, which included a range of psychopathology, was significantly higher than the paternal risk, which comprised primarily OCD and anxiety disorders. Further work will be needed to clarify whether results reflect maternal-specific environmental risks, genetic risks, factors related to parental care-seeking, or (to a modest extent, given the typical ages of onset for various parental disorders identified) parental stress.

***Genetics.*** A large mixed genetic sample yielded two heritable collections of symptoms that cross diagnostic boundaries, here named symmetry (including some other obsessions and compulsions) and disinhibition (including complex verbal tics) ( Darrow *et al.*, 2017b). Whole exome sequencing from over 500 trios identified a clear excess of likely gene-disrupting *de novo* mutations, and 4 risk genes that were altered by different mutations in multiple probands ( Willsey *et al.*, 2017). Another report described whole exome sequencing in a 3-generation family with TS, using induced pluripotent stem cells converted into neuronal cell types and assessing protein expression levels ( Sun *et al.*, 2018). The *PNKD* gene product was expressed at a lower rate in family members with TS or OCD.

### Pathophysiology

Roger Albin wrote a very thoughtful review of TS as “a disorder of the social decision-making network” ( Albin, 2018). Beste & Münchau (2018) describe tics in the context of the Theory of Event Coding, which describes the brain’s bidirectional pairing of stimulus and movement. They note the salience of urges to most tic patients, and the fact that most tics are individually relatively normal movements, but occur repeatedly and out of context. Similarly, Shafer *et al.* (2017) focus on the strong connections between movement and sensory function to propose a theory that encompasses both the development of stereotypies as part of typical development and their persistence in maladaptive forms.

***Animal models.*** A special issue on animal models of TS appeared in the Journal of Neuroscience Methods ( Bortolato & di Giovanni, 2017). Articles reviewed gait and sensorimotor function in the D1CT-7 mouse model of TS ( Fowler *et al.*, 2017), stress mediating the timing of abnormal movements in animal models ( Godar & Bortolato, 2017), and chemogenetic and optogenetic models ( Burton, 2017). Deer mice have behavior that has been discussed as a natural model of OCD ( Wolmarans *et al.*, 2018).

Recently postnatal ablation of the TrkB receptor in cells expressing parvalbumin was shown to produce dramatic changes in cortex and cerebellum and “profound hyperactivity, stereotypies, motor deficits and learning/memory defects” ( Xenos *et al.*, 2017). This result may help explain why autopsies in TS show a lower number of parvalbumin-containing interneurons in the striatum, but this model also produces much more substantial neuroanatomical changes than are seen in TS.

A gene identified in human OCD, *slc1a1*, which codes an excitatory amino acid transporter, was altered in mice to prevent its expression and function ( Zike *et al.*, 2017). The loss of this protein resulted in mice with reduced extracellular dopamine concentrations and reduced movement and stereotypic behavior after challenge with amphetamine or a dopamine D _1_ receptor agonist. Restoring the gene’s expression in the midbrain, but not in the striatum, partially rescued the exogenous dopamine-induced stereotypies. This research is important for its direct links to human illness and its anatomical specificity, and lends additional support to testing dopamine D _1_ antagonists in TS (see *Medication* section, below).

***New insights from computational modelling.*** Using the neurophysiological data obtained from a TS animal model of pharmacological striatal disinhibition, Caligiore *et al.* (2017) proposed a computational model of the basal ganglia-cerebellar-thalamo-cortical system to address the mechanisms of motor tic generation in TS. Overall, the model suggested that interplay between dopaminergic signal and cortical activity triggered the occurrence of a tic and was able to predict the number of tics generated when striatal dopamine increases and when the cortex is externally stimulated. Maia & Conceição (2017) further discussed the role of tonic and phasic dopamine in tics learning and expression. Based on the existing literature of habit formation and reinforcement learning in TS, the authors proposed a model of tics as exaggerated and persistent motor habits reinforced by aberrant, increased phasic dopamine responses. According to this model, tonic dopamine release would serve to amplify the tendency to execute learned tics. The authors also proposed the mechanism of antipsychotics’ action on tics: increased activity of indirect pathway due to antipsychotic administration could result in tic reduction, but at the same time potentially also could increase the propensity for reinforcing tics due to plasticity in the indirect pathway. In contrast, the authors also suggested that low-dose dopamine agonists could decrease both phasic and tonic dopamine and thus reduce both tic learning and tic expression. Both of these reports assume increasing tics with increasing dopamine concentrations, an assumption that seems to contradict observations that tics do not worsen with exogenous levodopa ( Black & Mink, 2000; Gordon *et al.*, 2013) nor improve with development of Parkinson disease ( Kumar & Lang, 1997; Martinez-Torres *et al.*, 2009; Shale *et al.*, 1986). Furthermore, low-dose pergolide in children and adolescents with TS that reduced tic severity *suppressed* prolactin rather than increasing it, consistent with overall enhancement of dopamine transmission, at least in the hypothalamic-pituitary dopaminergic pathway ( Gilbert *et al.*, 2000a; Gilbert *et al.*, 2000b).

Based largely on available functional anatomical studies, Conceição *et al.* (2017) also proposed a computational model of premonitory urges in TS. According to this model, premonitory urges and in particular their termination, like termination of other aversive stimuli, might elicit positive prediction errors, supported by phasic dopamine release that would then reinforce tics. The insula may play a central role in aversive feeling associated with premonitory urges and their learned negative value. The insula might send this information via direct or indirect projections to dopamine neurons, which might use it for calculation of the positive prediction errors that occur with termination of the premonitory urge. In short, the authors provide a more detailed neurobiological explanation for the classic model that premonitory urges may strengthen tics through negative reinforcement.

***Cognitive function and decision-making in TS.*** Morand-Beaulieu *et al.* (2017a) provide an updated and exhaustive review of neuropsychological aspects of TS (compare Eddy *et al.* (2009)). The review highlighted the slight alteration of social cognition as well as more frequent learning difficulties and disabilities in children with TS. Recent data also seem to confirm the deficit in executive function in TS as indexed by poor performance in continuous performance test and Stroop tests. Interestingly, using longitudinal evaluation of executive function in children with TS, Yaniv *et al.* (2018) showed that adults with TS showed response inhibition deficits, that tic reduction over time was significantly associated with development of response inhibition, and that former TS patients whose tics had remitted performed as well as, or on some tests better than, healthy control subjects. In contrast, the attentional and memory capacity seems to be impacted by comorbid symptoms more than TS *per se*. Sample size (n=122) or the healthy control group may explain different results obtained by Abramovitch *et al.* (2017), who studied treatment response in TS. They concluded that “the finding that significant change in symptom severity of TS/CTD patients is not associated with impairment or change in inhibitory control regardless of treatment type suggests that inhibitory control [as measured by the tests selected] may not be a clinically relevant facet of these disorders in adults.” Given these contradictory results, Morand-Beaulieu *et al.* (2017b) review the puzzling question of inhibitory control in TS in a recent meta-analysis, and find larger inhibitory deficits in TS + ADHD patients, but this deficit was also present in “pure” TS. This deficit in TS was most prominent in verbal responses, was associated with tics severity as assessed with Yale Global Tic Severity Scale-Total Tics Score (YGTSS-TTS) and was larger in studies that included medicated TS patients.

Salvador *et al.* (2017) study decisional capacities in TS and specifically the ability to learn from the outcomes of alternative courses of action (known as counterfactual learning). Unmedicated patients with TS showed normal performance on this task, whereas alteration was found in TS patients treated with the dopamine D2-like receptor (D2R) partial agonist aripiprazole, suggesting that modulating D2Rs may impair certain aspects of human reinforcement learning.

The Committee on Research of the American Neuropsychiatric Association published a systematic review on the neurobiology of the premonitory urge in TS ( Cavanna *et al.*, 2017).

***Electrophysiology.*** Brandt *et al.* (2017) reported on enhanced multi-component behavior in TS, which was also reflected in a smaller P3 event-related potential measured by EEG and potentially related to chronic tic control in these patients. An EEG study during simulated driving found that “mind wandering” may be quantifiable using EEG measures of alpha power and the P3a component of an auditory event-related potential ( Baldwin *et al.*, 2017). Since mind wandering is a defining feature of ADD, in addition to being ubiquitous during repetitive tasks, these measures may prove useful in studying ADHD phenomenology in TDs.

***Neuroimaging studies.*** Polyanska & colleagues. (2017) provide a meta-analysis of task-based neuroimaging reports in TS. Adults with TS show some impairment in lateralized sequential finger tapping movements, and this impairment was compared to fractional anisotropy of white matter tracts connecting primary motor cortex (M1) to M1 and supplementary motor area (SMA) to SMA ( Martino *et al.*, 2017a).

Previous *in vivo* MRI studies of basal ganglia volume and shape in TS have produced differing results (reviewed in Greene *et al.* (2017)). A study of 47 children age 8-12 with TS, and controls with or without ADHD, found no significant group differences ( Forde *et al.*, 2017).

A PET study of the microglial activating marker TSPO in OCD found elevated TSPO concentrations in dorsal caudate, orbitofrontal cortex, thalamus, ventral striatum, dorsal putamen, and anterior cingulate cortex ( Attwells *et al.*, 2017). Concentrations in patients were about 1/3 higher than in controls. This report implicates low-level brain inflammation in the pathophysiology of OCD.

***Clinical and neuropsychological studies.*** A study using the alternating serial reaction time task found no impairment of procedural learning in TS or ADHD ( Takács *et al.*, 2017). This result is surprising, given that habit learning on a “weather prediction” task is slower in TS ( Kéri *et al.*, 2002; Marsh *et al.*, 2004), but may indicate that the two tasks engage different learning systems, a conclusion supported by the generally normal cognitive function in TS.

Münchau and colleagues extended their previous work on echopraxia (imitation) to children with TS ( Brandt *et al.*, 2017). Participants were asked to lift either their index or pinky finger when prompted by an auditory tone; simultaneously they were shown a compatible or incompatible visual stimulus. Children with TS were slower overall but thereby gained less interference from the incompatible stimulus. The authors conclude that these results suggest that children with TS may employ “different or additional inhibition strategies” than children without tics. Again, on this task children with TS show superior, not deficient, action inhibition.

### Treatment

Ganos *et al.* (2017) review treatments for TDs in children.

***Psychological interventions.*** Houghton *et al.* (2017) analyzed their existing data to test the longstanding theory that habituation to premonitory urges during adequate periods of tic suppression is the mechanism by which behavior therapies for tic disorders exerted their beneficial effects. In two previous randomized trials, 126 children and adolescents and 122 adults with TS or a persistent TD had been assigned to Comprehensive Behavioral Intervention for Tics (CBIT) or to education and supportive therapy. The prediction was that urge severity would decrease in CBIT responders. Surprisingly, however, children showed no significant reduction in premonitory urges with treatment, even though CBIT was quite effective in the child study, and declines in urges in adults had no association with clinical improvement or group assignment. The authors conclude that habituation cannot be the underlying process by which CBIT exerts its beneficial effects. This important result warrants further study.

Another report from the same data set examined what clinical features at baseline predicted improvement in tics during treatment ( Sukhodolsky *et al.*, 2017). Importantly, those treated with CBIT improved regardless of medication status, while in the control (supportive therapy) group, tics improved only in those taking medication for tics. Also importantly for the understanding of behavior therapy in TS and patient selection, other psychiatric symptoms, age, sex, family functioning, and expectation of improvement had no significant effects on benefit from therapy. And contrary to some opinions, patients with worse tic severity had significantly *better* improvement in tics with CBIT. Anxiety disorders and, surprisingly, more severe premonitory urges predicted less improvement.

O’Connor and colleagues published a book describing their combined psychotherapeutic approach to tics ( O’Connor *et al.*, 2017).

Several groups reported efforts to increase dissemination and use of behavior therapy for tics. An internet-based, therapist-guided behavior therapy for tics is being studied in the BiP-TIC project ( Karlsson, 2016). The TicHelper.com internet-based CBIT program went live in late 2017 and received a positive review ( Conelea & Wellen, 2017).

***Medication.*** A group of international experts provided a status report and recommendations for using brain imaging for the rational development of novel psychopharmacological interventions ( Suhara *et al.*, 2017). As an example, a fascinating study in pediatric ADHD showed that fMRI response to a cognitive task (Go/No-Go) strongly predicted better clinical response to methylphenidate than to atomoxetine ( Schulz *et al.*, 2017). As the authors conclude, “These data do not yet translate directly to the clinical setting, but the approach is potentially important for informing future research and illustrates that it may be possible to predict differential treatment response using a biomarker-driven approach.”

Initial results from the first randomized, controlled trial (RCT) with a dopamine D _1_ receptor antagonist in pediatric TS were released by the sponsor in January, 2017 ( Chipkin, 2017). These new results supported the positive results from a pilot study in adults with TS ( Gilbert *et al.*, 2014), and suggest a novel treatment mechanism for tics.

In April, the US FDA approved the presynaptic dopamine depleting agent valbenazine (Ingrezza®) for treatment of tardive dyskinesia ( Neurocrine Biosciences, Inc. 2017b). TS is a likely off-label use for the drug, as the company has been conducting studies in children and adults with TS ( ClinicalTrials.gov). The FDA designated valbenazine an orphan drug for pediatric patients with TS ( Neurocrine Biosciences, Inc. 2017a). Another VMAT2 inhibitor, tetrabenazine, has been used for some time in the treatment of TS ( Marsden, 1973; Sweet *et al.*, 1974; Jankovic, 2016). A related compound, deutetrabenazine ( Paton, 2017), showed initial positive results in TS ( Jankovic *et al.*, 2016), and the FDA approved it for treatment of tardive dyskinesia in August ( Business Wire, 2017).

Aripiprazole has become a drug of choice in treating tics and comorbidities in TS over the past decade. However, large scale trials have been missing. Moreover, aripiprazole is not marketed for children and adolescents in many countries, regardless of the indication. Sallee *et al.* (2017) report on a phase 3, randomized, double-blind, placebo-controlled trial in 133 pediatric patients randomized in a 1:1:1 ratio to low-dose aripiprazole (5 mg/day if <50 kg; 10 mg/day if ≥50 kg), high-dose aripiprazole (10 mg/day if <50 kg; 20 mg/day if ≥50 kg), or placebo for 8 weeks. The primary efficacy endpoint was mean change from baseline to week 8 in the YGTSS-TTS. The Clinical Global Impression-Tourette’s Syndrome improvement score was also evaluated. High-dose aripiprazole was more effective than low-dose aripiprazole, and both were superior to placebo. Importantly, tolerance was overall good and no serious adverse events or deaths occurred, indicating that oral aripiprazole is a safe and effective treatment for tics in children and adolescents. These results provide important reassurance to clinicians who have been using aripiprazole for TS for years now. The placebo response rate (for the Clinical Global Impression scale), though half the response rate in the active treatment groups, was nevertheless surprisingly high (38%).

A small ( *N*=34) RCT of guanfacine showed no meaningful difference in effects on tic ratings or clinical impressions of improvement between the drug and placebo groups ( Murphy *et al.*, 2017). This result is important and surprising, given that adrenergic α2 agonists have been seen as first-line treatment for TS, especially in TS patients with ADHD ( Hollis *et al.*, 2016). Both these studies show how important RCTs are to clinical care in TS. Speaking of RCTs and guanfacine, a press release reported that extended-release guanfacine showed superiority to placebo in adults with ADHD (discussed here). The importance of this report comes primarily from the fact that data on ADHD treatments are scarcer in adults than in children.

Cannabinoids for TS are increasingly being studied; a brief summary of some of this work appears in Black (2017).

***Neurosurgery.*** A fascinating study demonstrated in mice that interfering electrical “beats” (similar to the beats one hears when tuning one instrument to another) can be used to steer neuron activation to focal sites in the brain without surgical electrode implantation ( Grossman *et al.*, 2017). Much work remains to be done to demonstrate feasibility, safety and efficacy in humans, but this approach potentially could lead to noninvasive, focal brain stimulation.

Welter *et al.* (2017) performed a randomized, double-blind, controlled trial of deep brain stimulation (DBS) of the posterior and anterior internal globus pallidus for severe TS. The design was very similar to that reported previously by Kefalopoulou *et al.* (2015), and so were the results. The primary endpoint was the difference in YGTSS score between the beginning and end of the 3 month double-blind period. No significant differences between groups were noted in YGTSS score change between the beginning and the end of the 3 month double-blind period, despite a slight improvement in the stimulated condition. However, after the end of the open stimulation period and further parameter adjustment, results become significant compared to baseline. Also, when turning the stimulators off in a blinded fashion, YGTSS scores increased again to reach near baseline levels. Overall, this study is most important in terms of optimal study design. The stimulator programming period preceding the blinded phases was likely too short and the parameters suboptimal (a choice intended to reduce unblinding). Future studies will need to consider these results carefully.

A London center reported an analysis of GPi DBS data, looking for the “sweet spot” for DBS for tic improvement ( Akbarian-Tefaghi *et al.*, 2017). They report that “a region within the ventral limbic GPi, specifically on the medial medullary lamina in the pallidum at the level of the AC-PC, was significantly associated with improved tics but not mood or OCB outcome.” Two patients with DBS to the fields of Forel experienced a good outcome ( Neudorfer *et al.*, 2017).

TS patients undergoing DBS, independent of the surgical target, usually require high stimulation parameters, leading to short (2–3 years) battery life, meaning frequent and costly battery replacements. Michael Okun and colleagues showed proof of the principle that triggered rather than continuous DBS may be helpful ( Molina *et al.*, 2017). In a single patient with medically refractory TS, a spectral feature in the 5- to 15-Hz band was used as the control signal from bilateral leads in the centromedian-parafascicular (Cm-Pf) region of the thalamus. Significant tic improvement compared to baseline was observed 12 months after the procedure, similar to that obtained with continuous DBS, and resulted in a 63% improvement in the neurostimulator’s projected mean battery life. These so called closed-loop systems are gaining traction in various CNS diseases where DBS is applied, and this case report paves the way in TS.

A report from the DBS group in the Netherlands called attention to side effects over the course of treatment in TS patients with thalamic DBS ( Smeets *et al.*, 2018). A subthalamic nucleus (STN) DBS study in OCD reminds us that DBS can cause side effects; STN stimulation at higher voltages caused chorea-ballismus ( Mulders *et al.*, 2017).

### Tics, family and society

Quality of life is lower in parents of children with TS and is more related to factors other than tic severity ( Jalenques *et al.*, 2017). Stewart *et al.* (2015) previously reported similar results. These studies emphasize the need to assess and treat symptoms other than tics in TS patients, and to care for the whole patient.

## Conclusions

2017 has again seen a rise in publications on TS, reflecting the increased interest this field receives, both from clinicians and researchers devoted to this disorder but also from adjoining fields, given the substantial psychopathology associated with TDs.

One important question raised is the natural course of TS and the rate of (non-)remission. Recent data suggest that the optimistic outlook prevalent with regard to tic severity when gliding from adolescence into adulthood might not be completely justified. Here, large longitudinal studies are warranted, and also to better understand the prognostic factors associated with tic remission or persistence. The fate of comorbidities also needs to be better understood, even though data have begun to emerge. We also still face important delays in the diagnosis of TS, and we need to deal with the recently demonstrated increased suicide risk in this condition, which underlines that TDs are far from benign.

Of note, there is a rise in computational models of TS, which provide important leads to understanding its pathophysiology and will likely fuel more targeted treatment approaches for tics. In the therapy field, 2017 has mainly seen studies centered on well-known pharmacological targets such as the dopaminergic system; however, new antidopaminergic medications were studied or came to the market in 2017, and increasing data supports existing off-label prescription practices. Behavioral therapy continues to emerge as a main pillar of tic treatment, with an improved understanding of its mechanisms and response factors. In the surgical field, deep brain stimulation for severe TS cases continues to draw interest; in particular, target choice, length of stimulator programming for optimal outcome, and closed-loop systems are under active investigation.

Overall, the field is active and burgeoning. Breakthroughs are to be expected in the upcoming years, especially with regard to large-scale efforts in the field.

## Data availability

No data are associated with this article.
