# Supplementary material for: Tourette syndrome research highlights from 2017
Source: F1000Res. 2018 Jul 23;7:1122. [Version 1] doi: 10.12688/f1000research.15558.1 (PMC6107994; doi:10.12688/f1000research.15558.1)
Supplement: Peer review history

# Peer review: Referee response for version 1

**DOI:** 10.5256/f1000research.16971.r37057

**Published:** 2018-09-10

**Authors:** Thomas Fernandez

**Affiliations:** 1 Child Study Center, Department of Psychiatry, Yale University, New Haven, CT, USA

This is a brief review of the literature pertaining to Tourette Syndrome in 2017. This review appears to include the main studies from 2017, summarizing them accurately. They provide a conclusion section that highlights what the authors believe are the most important themes in research for 2017 and important areas to investigate for 2018. Overall, I feel that this is a nice way for researchers in the field to quickly come up to speed with what is being done in TS research, and a good collection of references for closer review.

I have read this submission. I believe that I have an appropriate level of expertise to confirm that it is of an acceptable scientific standard.

# Peer review: Referee response for version 1

**DOI:** 10.5256/f1000research.16971.r37059

**Published:** 2018-08-23

**Authors:** Lorena Fernández de la Cruz

**Affiliations:** 1 Department of Clinical Neuroscience, Centre for Psychiatry Research, Karolinska Institutet, Stockholm, Sweden

This is a very well written, concise, and useful manuscript summarising the literature published in TS during 2017. I commend the authors’ efforts and I am grateful for the opportunity to review this piece. I only have a few minor comments, listed below.

 1. The choice of articles is obviously subjective, but I think that the review could also cite the following register-based study from Finland, including 1195 cases, about parental age and the (lack of) association with tic disorders. I believe that the study is relevant from an etiological point of view since it contrasts with what it is known in other disorders (e.g., ASD, ADHD):

 Chudal R, Leivonen S, Rintala H, Hinkka-Yli-Salomäki S, Sourander A (2017) ^1^

 2. Regarding the Fernández de la Cruz et al. (2017b) paper on suicide, cited in the Epidemiology section, the authors write: “TDs in adults were associated with a four-fold higher risk of suicide…” I suggest that the authors delete “in adults”. This large epidemiological cohort included individuals of all ages, followed up for different periods of time, up to 44 years, and the outcomes may have occurred at any time during the follow-up, including childhood/adolescence.

 3. When mentioning the paper by Darrow et al. (2017a), the authors explain: “The presence of OCD was associated with higher scores on the social cognition and RRB subscales”. I assume that a higher score in the RRB subscale means more repetitive behaviours, but does an elevation in the social cognition subscale reflect more (better) or less (worse) social cognition? I take from the following sentence that it is probably worse, but please clarify.

 4. When referring to the BIP TIC project in the Psychological interventions section, you may want to cite the communication cited below, which was presented at the ESSTS conference in 2017, and/or the project’s registration ( https://clinicaltrials.gov/ct2/show/NCT02864589) instead of the Karlsson, 2016 reference.

 Andrén, P. (2017, June). *Development and evaluation of a therapist-guided, Internet-based behaviour therapy programme for young people with Tourette syndrome and chronic tic disorder: The BIP TIC programme.* 10th European Conference on Tourette Syndrome and Tic Disorders / 2017 Annual Meeting of the European Society for the Study of Tourette Syndrome, Seville, Spain. [oral and poster presentation]

 Other minor points:

- The acronym ADD is only used once in the text (in the Electrophysiology section) and is never spelled out. Please write “attention deficit disorder” instead.
- Please spell out TSPO the first time that is mentioned (Neuroimaging studies section).

I have read this submission. I believe that I have an appropriate level of expertise to confirm that it is of an acceptable scientific standard.

## References

[1] ChudalRLeivonenSRintalaHHinkka-Yli-SalomäkiSSouranderA: Parental age and the risk of obsessive compulsive disorder and Tourette syndrome / chronic tic disorder in a nationwide population-based sample. *Journal of Affective Disorders*.2017;223: 10.1016/j.jad.2017.07.033 101-105 10.1016/j.jad.2017.07.033 28738196

# Peer review: Referee response for version 1

**DOI:** 10.5256/f1000research.16971.r36381

**Published:** 2018-08-06

**Authors:** Keith A. Coffman

**Affiliations:** 1 Division of Pediatric Neurology, Department of Pediatrics, Children's Mercy Hospital, Kansas City, MO, USA

I thoroughly enjoyed this manuscript, except for two paragraphs. 

 First, on Page 2, right column, the following sentence should be incorporated into another paragraph or eliminated as it is awkward standing alone.

 "Martino et al. (2017c) review screening instruments and rating scales for TDs; see also (Augustine et al.2017; Martino &

 Pringsheim, 2017)."

 Second, on Page 5, the paragraph that starts, "A small (N=34) RCT of guanfacine showed no meaningful difference in effects on tic ratings or clinical impressions of

 improvement between the drug and placebo groups (Murphy et al., 2017)" is incorrect. The study that Murphy and colleagues did was using EXTENDED RELEASE guanfacine, which has significantly less bioavailability than immediate release quanfacine. The point of their study was that the extended release preparation was ineffective, not that guanfacine was ineffective.

I have read this submission. I believe that I have an appropriate level of expertise to confirm that it is of an acceptable scientific standard.

# Response

**Published:** 2018-08-06

**Authors:** Kevin J Black

**Affiliations:** Department of Psychiatry, Washington University in St Louis, USA

Thanks for the thoughtful review. We can address these points in the revision after we receive comments from other reviewers.
